# Viviparity stimulates diversification in an order of fish

**DOI:** 10.1038/ncomms11271

**Published:** 2016-04-12

**Authors:** Andrew J. Helmstetter, Alexander S. T. Papadopulos, Javier Igea, Tom J. M. Van Dooren, Armand M. Leroi, Vincent Savolainen

**Affiliations:** 1 Department of Life Sciences, Silwood Park Campus, Imperial College London, Ascot, Berkshire SL5 7PY, UK; 2 Department of Conservation Science, Royal Botanic Gardens, Kew, Richmond TW9 3AB, UK; 3 Department of Plant Sciences, University of Cambridge, Downing Street, Cambridge CB2 3EA, UK; 4 Department of Evolutionary Ecology, CNRS/UPMC/UPEC/UPD/IRD/INRA, UMR 7618 Institute of Ecology and Environmental Sciences Paris (iEES), Université Pierre et Marie Curie, Case 237, 7 Quai St Bernard, 75005 Paris, France; 5 CNRS/ENS. UMS 3194, CEREEP. Ecotron IleDeFrance, École Normale Supérieure, 78 rue du Château, 77140 St-Pierre-lès-Nemours, France; 6 Research Team Endless Forms, Naturalis Biodiversity Center, Darwinweg 2, Leiden 2333 CR, The Netherlands

## Abstract

Species richness is distributed unevenly across the tree of life and this may be influenced by the evolution of novel phenotypes that promote diversification. Viviparity has originated ∼150 times in vertebrates and is considered to be an adaptation to highly variable environments. Likewise, possessing an annual life cycle is common in plants and insects, where it enables the colonization of seasonal environments, but rare in vertebrates. The extent to which these reproductive life-history traits have enhanced diversification and their relative importance in the process remains unknown. We show that convergent evolution of viviparity causes bursts of diversification in fish. We built a phylogenetic tree for Cyprinodontiformes, an order in which both annualism and viviparity have arisen, and reveal that while both traits have evolved multiple times, only viviparity played a major role in shaping the patterns of diversity. These results demonstrate that changes in reproductive life-history strategy can stimulate diversification.

## Abstract (web summary)

Live birth and an annual life cycle potentially enable access to new ecological niches and subsequent species diversification. Here, Helmstetter *et al.* build the phylogeny for fish in the order Cyprinodontiformes and find that, though live birth and annualism have each evolved multiple times, only live birth is associated with increased diversification.

## Graphical abstract

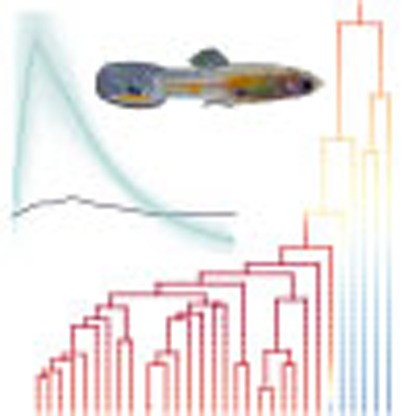

The rate at which a clade accumulates species depends on the balance between speciation and extinction. In recent years, phylogenetic studies have shown that such net diversification rates vary hugely. Where the coelocanth genus, *Latimera*, has produced only two known species in the past 80 million years^1^, the Haplochromine cichlids of Lake Victoria may have produced as many as 500 species in as few as 15,000 years^2^. Increases in net diversification rate are often thought to be driven by the evolution of novel phenotypes that provide access to new ecological niches^3^. However, to date, there have been few convincing examples of such phenotypes^4^,^5^. To demonstrate the effect of a proposed phenotypic trait on the rate of diversification, it is necessary to show that the evolution of this trait is repeatedly and independently associated with a change in net diversification rate^6^. Using the order Cyprinodontiformes, we tested the hypothesis that two reproductive life-history traits, viviparity and annualism, can drive rapid diversification.

The Cyprinodontiformes are an order of ∼1,250 ray-finned fish species found primarily in Africa and the Americas. Many of these species are popular in the aquarium hobby, including guppies, mollies and killifish. Living in a wide range of habitats, they have also evolved many different life-history strategies. Most Cyprinodontiformes have external fertilization and are oviparous, but∼27% of species have internal fertilization and are viviparous or ovoviviparous, which we group together for the remainder of this study and refer to as viviparity^7^. Their most remarkable life-history strategy, however, is annualism. Annual species are typically found in seasonal pools and wetlands on the African and South American grasslands, savannas and forests. When ponds dry out the adults die, but their embryos, which are buried and protected from desiccation by a thick chorion and an embryonic diapause, survive to hatch during the next wet season^8^. About 25% of Cyprinodontiformes are annuals, while the rest may live and breed for multiple years.

Both traits have the potential to affect diversification in different ways. Viviparity can allow for increased colonization rates and establishment by single gravid females, providing access to new geographic regions^9^. This may lead to geographic isolation eventually giving rise to speciation. Annualism provides access to new niche space previously unoccupied by fish species, that is, seasonal ponds. Following the colonization of this new habitat, geographic isolation and adaptation may drive bursts of speciation. Furthermore, the adaptations that enable survival in a seasonal system may act as a buffer against extinction. As a result, we predicted that these extraordinary reproductive life-history traits could be associated with increases in diversification rate.

To investigate this, we first built a generic level phylogenetic tree of Cyprinodontiformes, which we used to identify shifts in the rate of net diversification across the order. Next, we reconstructed ancestral states for both viviparity and annualism to determine when and how often these traits evolved. We then examined whether previously identified diversification rate shifts coincide with the evolution of annualism or viviparity. Finally, we determined whether annual or viviparous clades had increased diversification rates relative to the remainder of Cyprinodontiformes.

We find that viviparity triggered a burst of speciation in two out of the three groups in which it has evolved; furthermore, we find that viviparous species generally have a higher diversification rate than oviparous species. In contrast, annualism did not stimulate diversification.

## Results

### Phylogenetic analysis

**Figure 1:**
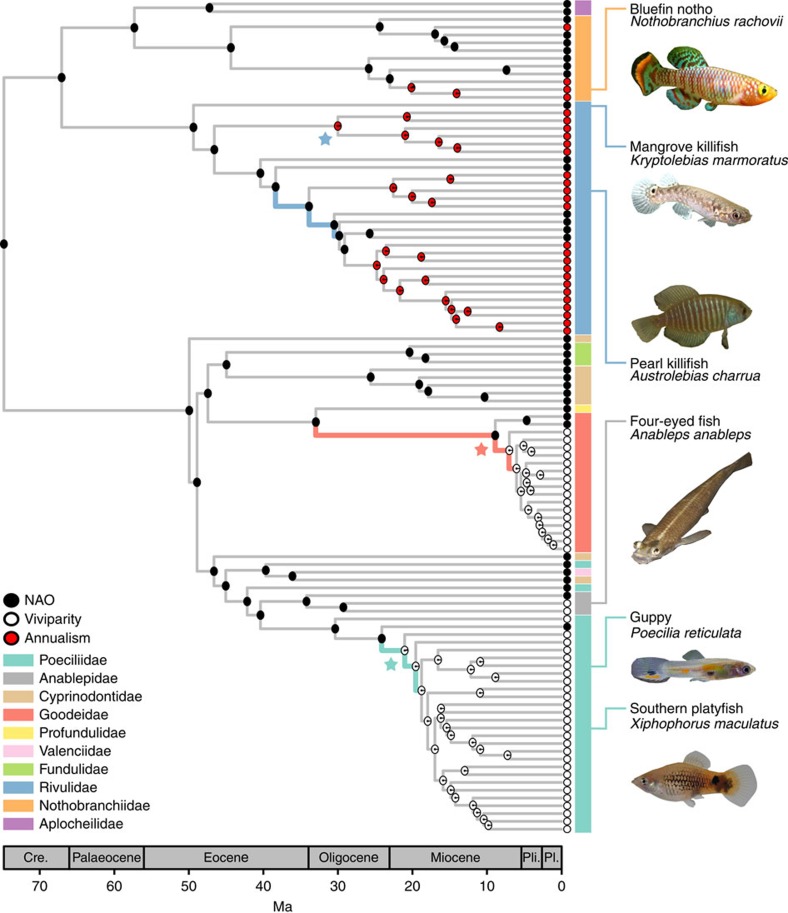
Phylogenetic tree of Cyprinodontiformes. A Bayesian maximum clade credibility tree is shown with ancestral reconstructed states of viviparity and annualism. Pie charts in each node represent MuSSE reconstructed ancestral states (NAO, non-annual oviparous). Branches on which BAMM indicated large support for rate changes (Bayes factor >20) are highlighted in colour. Stars denote the node from which clade diversification rates over time were calculated (shown in Fig. 2). Fish images used, except *Austrolebias charrua* (taken by TJMVD), were modified from images attributed (from top to bottom) to Cisamarc (CC BY-SA 4.0), Cardet co6cs (CC BY-SA 3.0), J.C. Harf (CC BY-SA 3.0), Per Harald Olsen (CC BY 3.0) from Wikipedia and The Xiphophorus Genetic Stock Center, respectively.

Using DNA sequence data and Bayesian inference, we produced a time-calibrated molecular phylogenetic tree of Cyprinodontiformes for 107 genera (Supplementary Fig. 1). This tree was well resolved with strong support (posterior probabilities>0.9) for 70% of the nodes (Supplementary Fig. 2). Our tree is broadly consistent with previously published phylogenetic trees of a subclade of Poeciliidae^10^, the family Goodeidae^11^ and suborder Apolcheiloidei^12^. All currently accepted Cyprinodontiform families were monophyletic except Cyprinodontidae and Poeciliidae (Fig. 1), whose taxonomy may, therefore, need to be re-evaluated. These taxonomic uncertainties do not affect our estimates of branching times and diversification rates.

### Modelling diversification rates

To model the dynamics of speciation and extinction on this phylogenetic tree, we used Bayesian analysis of macroevolutionary mixtures (BAMM)^13^. This analysis revealed several shifts in diversification rates within Cyprinodontiformes (Fig. 1; Supplementary Fig. 3). We found strong support for more than one diversification rate across the order; the posterior probability that 2–4 rate shifts have occurred was 0.96. Two primary shifts were recovered consistently in the posterior distribution of shift sets produced by BAMM (Supplementary Fig. 4). The first of these shifts was located at the base of Goodeidae, and the second in Poeciliidae. We calculated branch-specific Bayes factors under a model imposing a rate shift for a particular branch versus a model without that shift, and detected nine branches where there was strong support for a rate shift. Three of these branches were located within Rivulidae, three at the base of Goodeidae and three at the base of the live-bearing Poeciliidae, highlighting three regions where rate shifts are likely to have occurred (Fig. 1). Overall, we found considerable rate variation over the evolutionary history of Cyprinodontiformes for which we can attempt to uncover the underlying causes.

### Ancestral-state reconstruction

We then used ancestral state reconstruction (ASR) to determine when and how often annualism and viviparity have evolved in Cyprinodontiformes. ASR methods are often biased towards traits that are associated with increased diversification, preferentially assigning the node state to the trait associated with increased diversification^14^. To circumvent this issue, we reconstructed traits using a Multi-State Speciation and Extinction (MuSSE) model^15^. MuSSE reconstructions revealed that both viviparity and annualism (Fig. 1) have each evolved in five independent instances. ASR using stochastic character mapping agreed with MuSSE methods, except in Anablepidae and Poeciliidae, where there was substantial uncertainty regarding the origin of viviparity (Supplementary Figs 5 and 6). MuSSE reconstructions were considered more reliable because of their ability to cope with differential diversification rates and lack of uncertainty when reconstructing character state. They were therefore used in all subsequent analyses.

### Timing of diversification rate shifts

**Figure 2:**
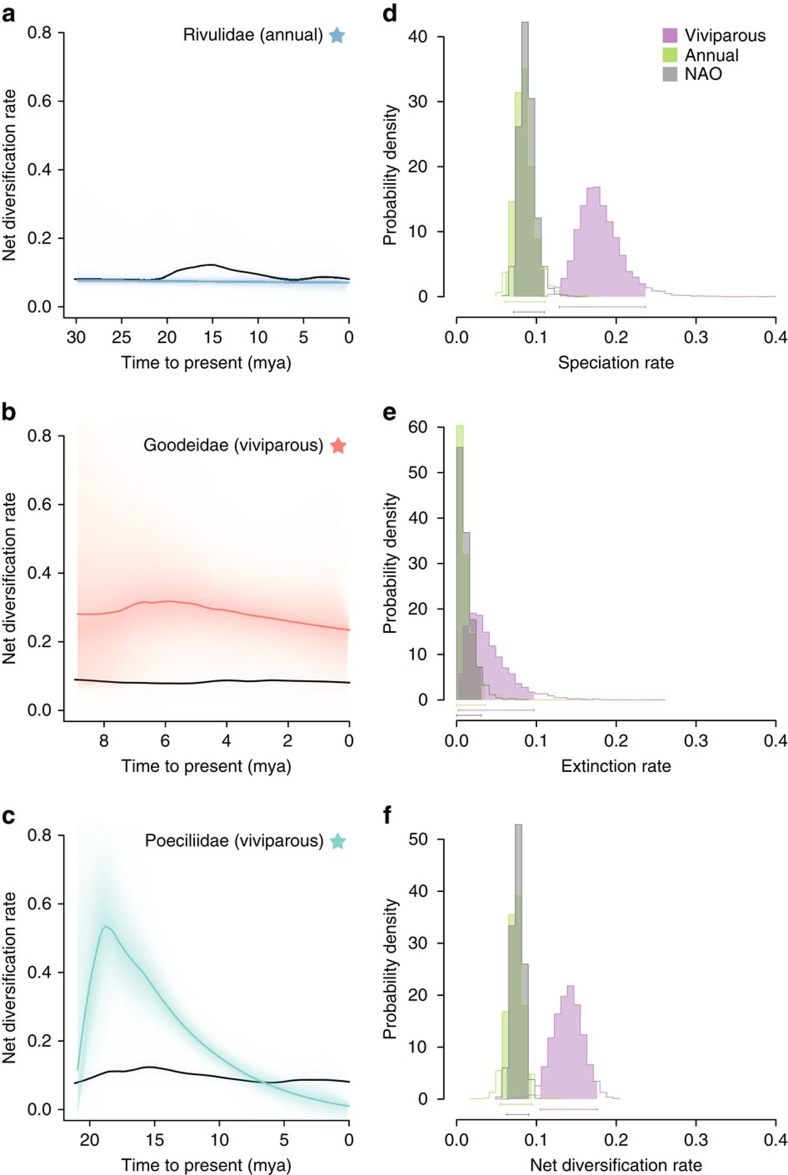
State-dependent diversification rates and rates over time in Cyprinodontiformes. Graphs on the left show net diversification rates over time for three clades in which annualism or viviparity evolved: (**a**) a clade of annual Rivulidae, (**b**) Goodeidae and (**c**) a clade of viviparous Poeciliidae (see stars in Fig. 1 and text for details). Coloured lines indicate net diversification rates against background rate (black lines), with shading around coloured lines representing 90% confidence intervals. On the right, state-dependent diversification rates extracted from BAMM analyses are shown for non-annual viviparous, annual oviparous and non-annual oviparous (NAO) clades. These graphs show the posterior distribution of rates for speciation (**d**), extinction (**e**) and net diversification (**f**), coloured by character state.

If the reproductive life-history traits have caused an increase in diversification, we would expect rate shifts to occur when or shortly after a trait has appeared. Combining our ASR and BAMM analyses, we found that the evolution of viviparity coincides with the sharp increase in diversification rates seen in Goodeidae and Poeciliidae, while the evolution of annualism did not overlap with increases in diversification (Fig. 2a–c). The rate shift identified in Poeciliidae was consistently placed at or adjacent to the evolution of viviparity in Poeciliidae, as shown in credible shift sets (Supplementary Fig. 4). Modelling diversification rates for the oviparous Empetrichthyinae, a subfamily within Goodeidae, proved difficult as BAMM analyses produced a bimodal distribution of rate estimates (Supplementary Fig. 7). Regardless, cumulative shift probabilities (the probability that rate shift has occurred between a given branch and the root) indicate that the best support for a rate different to the background rate lies immediately after the evolution of viviparity in Goodeidae and Poeciliidae (Supplementary Fig. 8). In addition, the highest branch-specific Bayes factors support a rate shift occurring on the branch leading to the viviparous Goodeidae (on which viviparity likely evolved) and the branch following the evolution of viviparity in Poeciliidae. These results confirm our expectation that diversification increased when or shortly after viviparity appeared and show that viviparity has been instrumental in stimulating diversification in these clades. Rate shifts did not overlap with the evolution of annualism in any cases and we therefore conclude that the evolution of annualism has had no causal effect on diversification shifts.

### Trait-dependent estimates of diversification

Finally, we looked for significant associations between character states and lineage-specific diversification rates using the posterior distribution of state-dependent rates from our BAMM analyses. We separated taxa into the three groups depending on character state: non-annual viviparous (viviparous), annual oviparous (annual) and non-annual oviparous (NAO). We found that net diversification and speciation rates in viviparous clades were approximately twice those of annual and NAO clades (Fig. 2d,f), while extinction rate did not differ between any groups (Fig. 2e). There were no significant differences between the diversification and speciation rates of annual clades and NAO clades (Fig. 2d,f). Significance was calculated by examining the posterior distribution of differences among the groups (Supplementary Fig. 9). We also modelled state-dependent rates using MuSSE^15^ and the output from these analyses were similar to the results derived from BAMM (see Methods; Supplementary Figs 10 and 11). At their maximum rate, viviparous lineages in families Goodeidae and Poeciliidae diversified up to three and five times faster than the background rate, respectively (Fig. 2b,c). The notable exceptions to the trend of higher diversification in viviparous clades were the genera *Anableps* and *Jenynsia*, which have diversification rates similar to the background rate (Supplementary Fig. 3). Regardless, the overarching pattern indicates that viviparity is associated with increased diversification rates.

## Discussion

So, how might viviparity promote diversification in freshwater habitats? As briefly mentioned in the introduction, one obvious way is that unlike oviparous fishes, viviparous females carry fertilized embryos with them. This means that a single, pregnant, viviparous female can colonize a new watershed, whereas a single, gravid, oviparous female cannot^9^; more frequent colonization of geographically isolated areas may facilitate speciation. There are other possibilities, for example, the young of viviparous species are also relatively protected from their environment^16^,^17^ and so may have higher survival rates than the young of oviparous species. This, in turn, might make the colonization of new habitats easier. Finally, viviparity allows for post-fertilization genomic conflicts to occur between mothers and embryos, siblings in the womb, and maternal and paternal genomes within embryos^18^. Such conflicts can perpetuate antagonistic coevolution, which may lead to increased post-zygotic reproductive isolation between populations and consequently stimulate speciation. Although we demonstrate a strong link between diversification and viviparity, we cannot disentangle these factors with our data, and future work should aim to discriminate between causal mechanisms. Unusually, the viviparous Anablepidae have diversification rates closer to oviparous taxa, and with our current data we cannot determine the cause of this low diversity. Further work could investigate whether factors, such as vicariance, dispersal, isolation or available niche space, have been notably limited in Anablepidae compared with the other viviparous groups.

Our finding that the evolution of viviparity has triggered multiple diversification rate shifts contrasts with results from reptiles. Viviparity has also evolved many times in reptiles^19^ where, contrary to our results, it has increased both rates of speciation and extinction, making net rates of diversification similar to those in the oviparous groups^20^. Future studies may expand on our work to examine the relationship between diversification and viviparity in sharks, amphibians and across all vertebrates.

We show that annualism has no effect on diversification rate, and rates were very similar to the NAO groups, which was surprising given our initial prediction that annualism buffers against extinction. One possible explanation is that annualism limits the maximum number of generations per year to one, thereby reducing mutation rate and speciation. Also, the ephemeral nature of seasonal ponds might limit the lifespan of populations and the speed at which advantageous life-history traits spread across them. Finally, we found that neither annualism nor viviparity has been lost during the evolutionary history of the Cyprinodontiformes. Again, this finding contrasts with evidence from reptiles, showing that transitions from viviparity to oviparity occur, though whether they are common^20^ or rare^21^ depends on the analytical approach.

We encountered some challenges in our study, and a number of caveats should be considered as a result. Although our study includes the replication necessary to link the evolution of a trait with a diversification rate shift, it is limited, with only two strong associations. MuSSE-like models are known to incorrectly estimate very low extinction rates^22^, which may have biased our estimates for annual species. This may also help to explain why our MuSSE analyses indicated that net diversification was significantly higher in annual clades than NAO clades, while speciation rates did not differ significantly. MuSSE-like models have received considerable criticism in recent years because of their low power at low sample sizes and heavy tip bias, and their susceptibility to type 1 errors^22^,^23^, so we take caution in interpreting our MuSSE results especially in regard to estimating extinction. Precise estimates of diversification rates require accurate dates for cladogenic events and are compromised to the extent that taxon sampling is incomplete. Although we have a near-complete generic level tree, it encompasses only 8.6% of Cyprinodontiform species. However, we are confident in our conclusions given that MuSSE and BAMM, which are independent methods that account for missing taxa in different ways, gave similar results (compare Fig. 2d with Supplementary Fig. 10a, Fig. 2e with Supplementary Fig. 10b and Fig. 2f with Supplementary Fig. 10c).

It is clear that the patterns of diversification will only be understood by interpreting our knowledge of organismal and life-history traits in view of the space and time patterns of environmental conditions encountered. For example, it may be that viviparity can only promote species diversity when combined with the variety and fragmentation typical of the freshwater habitats of Cyprinodontiformes. Regardless of these specific factors, our results demonstrate how the evolution of viviparity can have drastic effects on the distribution of diversity and go far towards explaining why so many species give birth to live young.

## Methods

### Phylogenetic analysis

Sequences from six nuclear genes and six mitochondrial genes were used to construct the phylogenetic tree of the Cyprinodontiformes. The nuclear genes used comprised of *enc1*, *pomgnt2*, *snx33*, *myh6*, *rag1* and *x-src*. The mitochondrial genes used included *CYTB*, *COX1*, *ND1*, *ND2*, *12S-rRNA* and *16S-rRNA*. *CYTB* was divided by codon position into separate alignments. Sequences for all genes for species assigned to the order Cyprinodontiformes were downloaded from Genbank (accessed September 2014) as well as sequences for seven outgroup species. We reduced the dataset to the longest sequence per gene per species. We then selected one species per genus that possessed sequences for the highest number of genes from our chosen set. The final data set included sequences from 85% of recognized Cyprinodontiform genera representing 94% of species. Sequences for all genes were aligned using the MAFFT (v1.3 Biomatters Ltd) plugin in Geneious v6.1.6 (ref. 24) using the auto alignment method after which the ends were trimmed, totalling up to 12,455 base pairs of sequence data. A table showing the sampled species and the loci used for each species can be found in Supplementary Data 1.

Sequences of 12 genes from 107 Cyprinodontiform species and 7 outgroup species were used to build a linked gene tree in BEAST v 1.8.0 (ref. 25). The appropriate nucleotide substitution model for each gene was determined using jModeltest v2.1.4 (ref. 26), using Akaike information criterion model selection. A relaxed lognormal molecular clock model was used for each of the genes, allowing substitution rates to vary between taxa. BEAST was run for 200 million generations and trees were sampled every 20,000 generations. Tracer v1.6 (ref. 25) was used to identify at which point stationarity had been reached and that the effective sample size (ESS) was >200 for all relevant parameters. We combined the logs and trees from three analyses using LogCombiner v1.8.0 (ref. 25), discarding the appropriate amount of burn-in for each analysis before combining. TreeAnnotator v1.8.0 (ref. 25) was then used to generate a maximum clade credibility (MCC) tree with the highest sum of posterior probabilities for all clades and target node heights. The tree was dated using five fossil calibrations and three secondary calibrations, leading to a total of eight calibrated nodes across the tree. Node ages and credibility intervals were taken from a recently published tree of bony fishes^27^ to date the nodes representing the most recent common ancestors of Cyprinodontiformes and Perciformes, Atheriniformes and Beloniformes. We used ages of fossils from the extant genera *Aphanius*^27^, *Fundulus*^28^,^29^, *Cyprinodon*^29^,^30^ and *Empetrichthys*^29^,^31^ and a single fossil from the extinct genus *Carrionellus*^32^. See Supplementary Table 1 for details of fossils and calibration points. Outgroup taxa were then pruned for all subsequent analyses.

### Ancestral-state reconstruction

Data for each trait were collected from FishBase^33^ (accessed September 2014) except in instances where information was not available and alternative sources were used (Supplementary Data 2). Using the MCC tree, we reconstructed ancestral states for character traits under a MuSSE model^15^. We divided the Cyprinodontiform genera into the following three character-state groups: non-annual viviparous, annual oviparous and NAO. Incomplete sampling was accounted for by a state-dependent sampling factor. We performed model simplification with MuSSE in a maximum likelihood framework based on likelihood ratio tests. Our simplification indicated that the simplest, best-fitting MuSSE model comprised of three speciation rates (one for each state), one extinction rate and one transition rate. The resulting MuSSE model and the full model were run in a maximum likelihood framework and the coefficients generated were used to perform ancestral-state reconstruction, resulting in almost identical outputs. We note that the MuSSE model indicates two origins of viviparity in a monophyletic clade of two Anablepidae genera. We believe this may be due to lower diversification rates in Anablepidae relative to other viviparous species. When accounting for increased diversification rate in viviparous species, MuSSE may overestimate the number of origins of viviparity in Anablepidae. Additional reconstructions using stochastic character mapping were performed with the make.simmap function in the phytools R package^34^ v0.4-31. This method fits a continuous-time reversible Markov model for the evolution of the selected trait. It then simulates stochastic character histories using that model and the tip states on the tree^34^. We simulated 1,000 character histories per trait, which produced results (Supplementary Figs 5 and 6) biased towards those traits associated with increase diversification rates as expected^14^. These results indicated a single origin of viviparity in Anablepidae. Finally, we performed ASR using the Binary State Speciation and Extinction model^35^,^36^ on viviparity and annualism independently, which produced identical results to the combined MuSSE reconstruction.

There is one well-known instance of variation in annualism in the genera shown on the phylogenetic tree, that is, in the genus *Fundulopanchax*^8^. In the analyses above, *Fundulopanchax* was scored as annual. However, we also reran all BAMM and MuSSE analyses, scoring this genus as non-annual, and it did not affect the results or plots of net diversification (Supplementary Fig. 12).

### Diversification rates

We used BAMM v2.2.0 (ref. 13) to estimate rates of speciation and extinction across the phylogeny of Cyprinodontiformes. BAMM allows variation in evolutionary rates through time and among lineages, relaxing the assumption that diversification rates must be time homogenous as in MEDUSA^37^. BAMM allows for the incorporation of incomplete taxonomic sampling at the backbone and clade level for which our tree contains 85% sampling of backbone taxa and varying proportions of species present sampling fractions per clade (Supplementary Data 2). We performed multiple BAMM runs of 30 million generations, sampling every 6,000 generations and checked convergence and stationarity using the CODA package v0.16-1 (ref. 38) in R. effective sample size (ESS) of all parameters were >200. The process was repeated three times to ensure convergence of separate runs. We then calculated the mean of the marginal posterior density of speciation, extinction and net diversification rates at all points on each branch of the summary tree. Credible shift sets, Bayes factor calculations and cumulative shift probabilities were obtained with the R package BAMMTools v2.02 (ref. 39). Speciation and extinction rates over time were calculated for each of the three trait groups (non-annual viviparous, annual oviparous and NAO) using all samples (minus burn-in) from the posterior distribution of a single run.

### Diversification correlates

We examined the differences of speciation, extinction and net diversification between viviparous, annual and NAO clades using the posterior distribution of state-dependent rates extracted from our BAMM analyses. We modified the getCladeRates() function of BAMMtools to select any required subset of nodes and tips to calculate a mean diversification rate across multiple clades of viviparous and annual species. We also calculated diversification rates for all NAO clades, including internal branches from our BAMM analyses. State-dependent rates were then compared using the credible intervals of differences to assess significance.

We verified these results using MuSSE and our MCC tree. MuSSE allows us to associate changes in speciation or extinction with multiple character states and has been developed to incorporate the effects of incomplete taxonomic sampling^15^. MuSSE was implemented using the R package Diversitree^15^. We fit a maximum likelihood (ML) MuSSE model with 12 parameters as a starting point to estimate parameters using a Bayesian framework. We again accounted for incomplete sampling using a sampling fraction for each state.

We then ran a MCMC chain of 10,000 generations using the full model and an exponential prior 1/(2*r*), where *r* is the character independent diversification rate. We removed 10% burn-in and then summarized the MCMC samples to assess variation in state-dependent speciation, extinction and net diversification rates (Supplementary Fig. 10). We calculated statistical significance between the character-state groups using the credible intervals of differences among posterior distributions of the state-dependent speciation, extinction and net diversification rates (Supplementary Fig. 11). These results supported the results derived from BAMM analyses, except in the case that net diversification rates of annual clades are higher than NAO clades

## Additional information

**How to cite this article:** Helmstetter, A. J. *et al.* Viviparity stimulates diversification in an order of fish. *Nat. Commun.* 7:11271 doi: 10.1038/ncomms11271 (2016).

## Supplementary Material

Supplementary Information Supplementary Figures 1-12, Supplementary Table 1 and Supplementary References

Supplementary Dataset 1 Sampled species, loci and accession numbers

Supplementary Dataset 2 Character states and sampling
