# Supplementary material for: Viviparity stimulates diversification in an order of fish
Source: Nat Commun. 2016 Apr 12;7:11271. doi: 10.1038/ncomms11271 (PMC4832061; doi:10.1038/ncomms11271)
Supplement: Supplementary Information — Supplementary Figures 1-12, Supplementary Table 1 and Supplementary References [file ncomms11271-s1.pdf]

Supplementary Figures

a

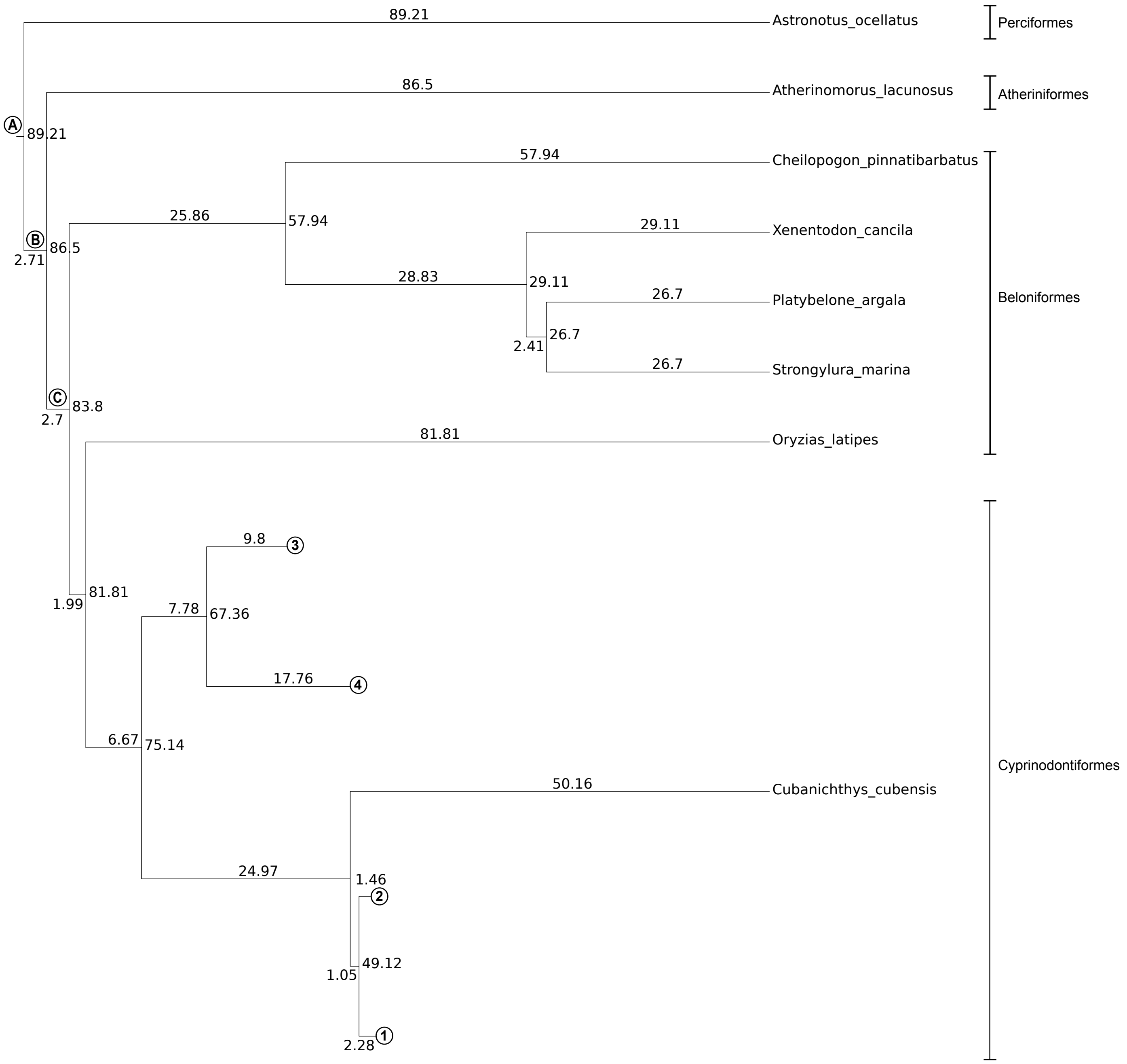

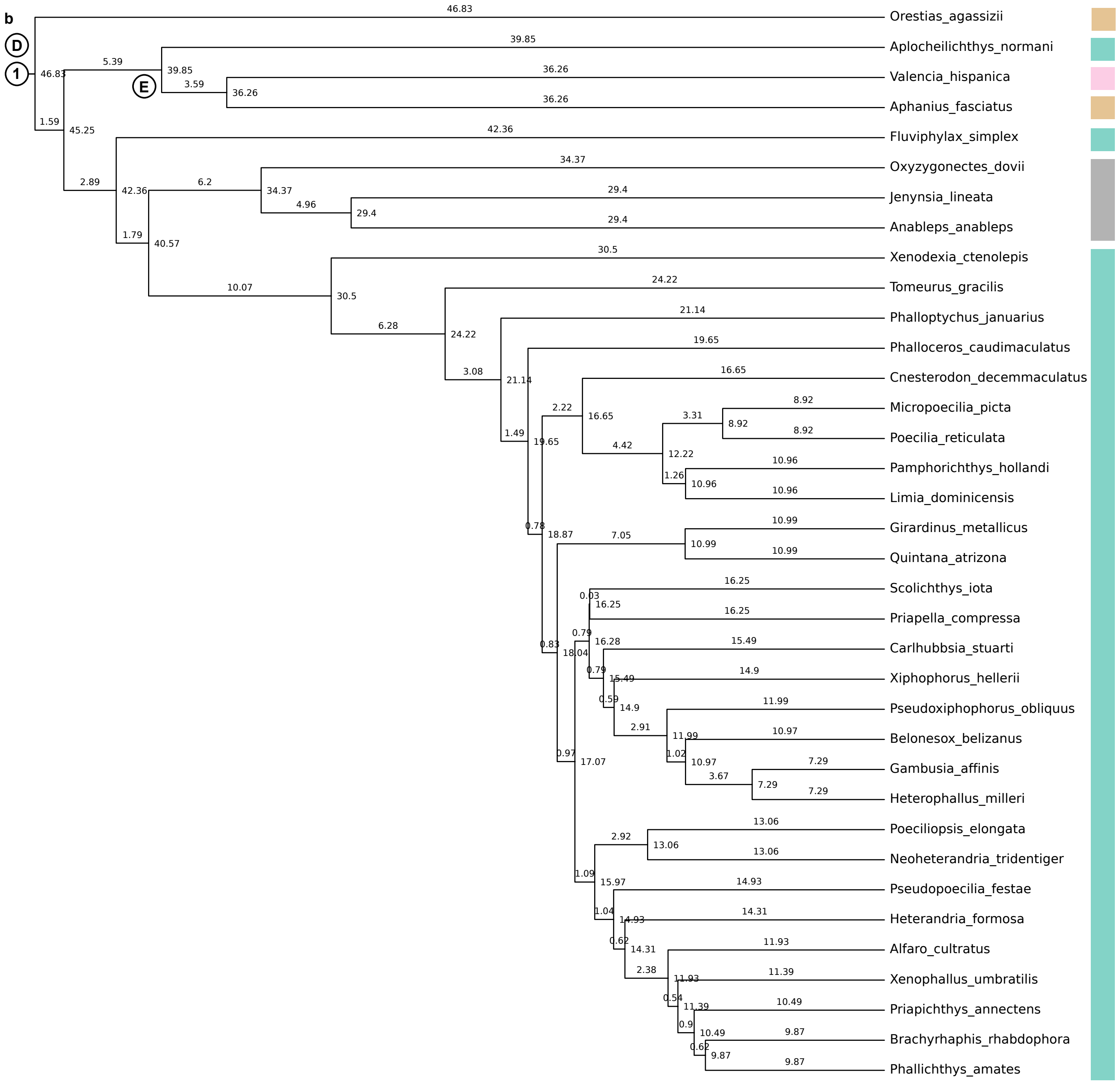
b
D
1
E
Orestias_agassizii
Aplocheilichthys_normani
Valencia_hispanica
Aphanius_fasciatus
Fluviphylax_simplex
Oxyzygonectes_dovii
Jenynsia_lineata
Anableps_anableps
Xenodexia_ctenolepis
Tomeurus_gracilis
Phalloptychus_januarius
Phalloceros_caudimaculatus
Cnesterodon_decemmaculatus
Micropoecilia_picta
Poecilia_reticulata
Pamphorichthys_hollandi
Limia_dominicensis
Girardinus_metallicus
Quintana_atrizona
Scolichthys_iota
Priapella_compressa
Carlhubbsia_stuarti
Xiphophorus_hellerii
Pseudoxiphophorus_obliquus
Belonesox_belizanus
Gambusia_affinis
Heterophallus_milleri
Poeciliopsis_elongata
Neoheterandria_tridentiger
Pseudopoecilia_festae
Heterandria_formosa
Alfaro_cultratus
Xenophallus_umbratilis
Priapichthys_annectens
Brachyrhaphis_rhabdophora
Phallichthys_amates

c

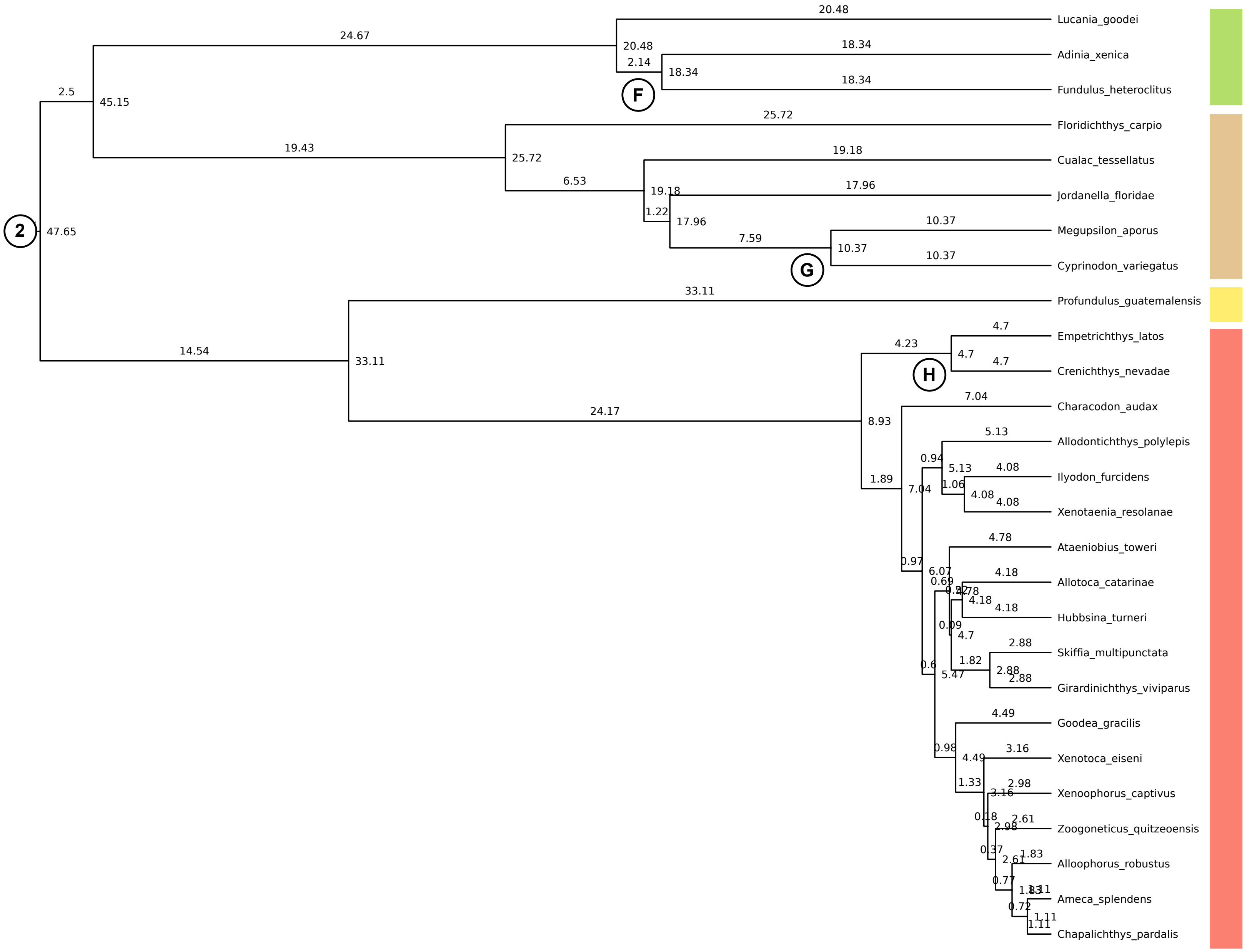
Lucania_goodei
Adinia_xenica
Fundulus_heteroclitus
Floridichthys_carpio
Cualac_tessellatus
Jordanella_floridae
Megupsilon_aporus
Cyprinodon_variegatus
Profundulus_guatemalensis
Empetrichthys_latos
Crenichthys_nevadae
Characodon_audax
Allodontichthys_polylepis
Ilyodon_furcidens
Xenotaenia_resolanae
Ataeniobius_toweri
Allotoca_catarinae
Hubbsina_turneri
Skiffia_multipunctata
Girardinichthys_viviparus
Goodea_gracilis
Xenotoca_eiseni
Xenoophorus_captivus
Zoogoneticus_quitzeoensis
Alloophorus_robustus
Ameca_splendens
Chapalichthys_pardalis
2
F
G
H
2.5
45.15
47.65
24.67
20.48
2.14
18.34
19.43
25.72
6.53
19.18
1.22
17.96
7.59
10.37
14.54
33.11
24.17
8.93
4.23
4.7
7.04
1.89
0.94
5.13
1.06
4.08
0.97
4.78
6.07
0.69
4.18
0.09
1.82
2.88
0.6
5.47
4.49
0.98
3.16
1.33
2.98
0.18
2.61
0.37
1.83
0.77
1.11
0.72

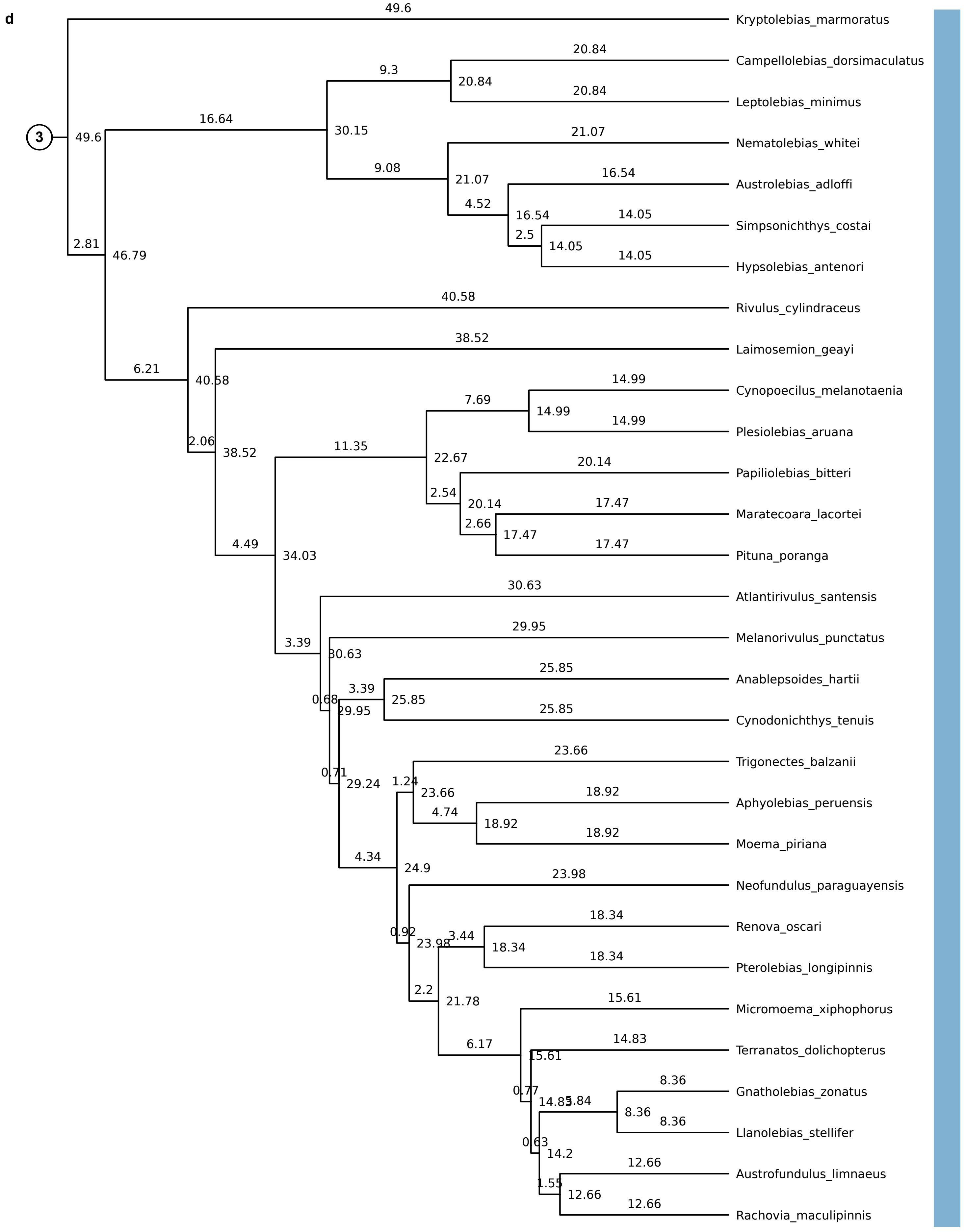
d
3
Kryptolebias_marmoratus
Campellolebias_dorsimaculatus
Leptolebias_minimus
Nematolebias_whitei
Austrolebias_adloffi
Simpsonichthys_costai
Hypsolebias_antenori
Rivulus_cylindraceus
Laimosemion_geayi
Cynopoecilus_melanotaenia
Plesiolebias_aruana
Papiliolebias_bitteri
Maratecoara_lacortei
Pituna_poranga
Atlantirivulus_santensis
Melanorivulus_punctatus
Anablepsoides_hartii
Cynodonichthys_tenuis
Trigonectes_balzanii
Aphyolebias_peruensis
Moema_piriana
Neofundulus_paraguayensis
Renova_oscari
Pterolebias_longipinnis
Micromoema_xiphophorus
Terranatos_dolichopterus
Gnatholebias_zonatus
Llanolebias_stellifer
Austrofundulus_limnaeus
Rachovia_maculipinnis

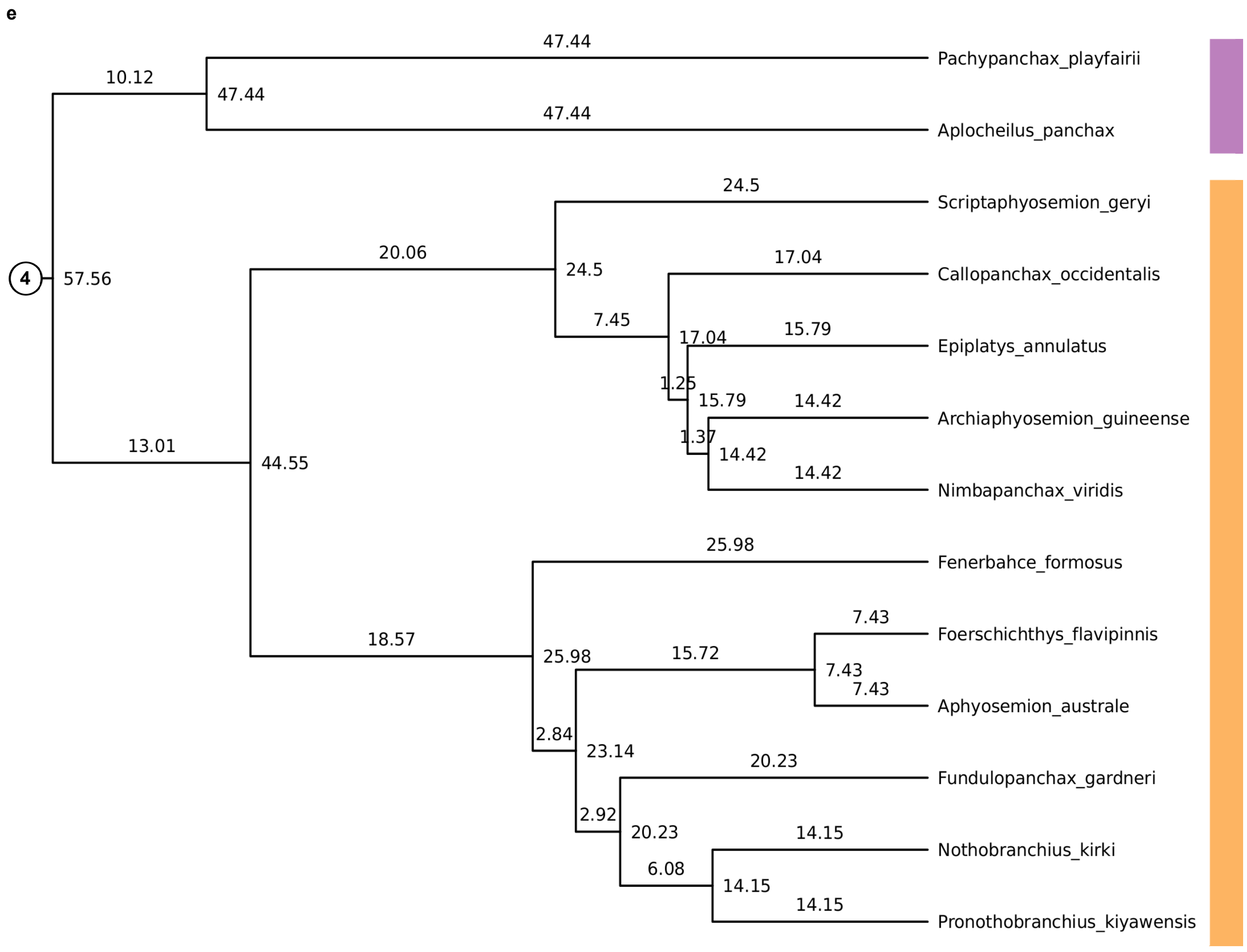

Supplementary Figure 1 | MCC phylogenetic tree split into subtrees. Branch lengths and node ages in Ma are shown. Panel **a** shows a skeleton tree with the position of each subtree (1, 2, 3, 4) labeled on the branch upon which it is found. The order each tip belongs to is shown on the right. Panels **b, c, d** and **e** show the subtrees where colours correspond to the family colour code used in Fig. 1. Tip labels show the species molecular data was taken from. Letters A to H indicate the nodes that were calibrated using fossils in Supplementary Table 1.

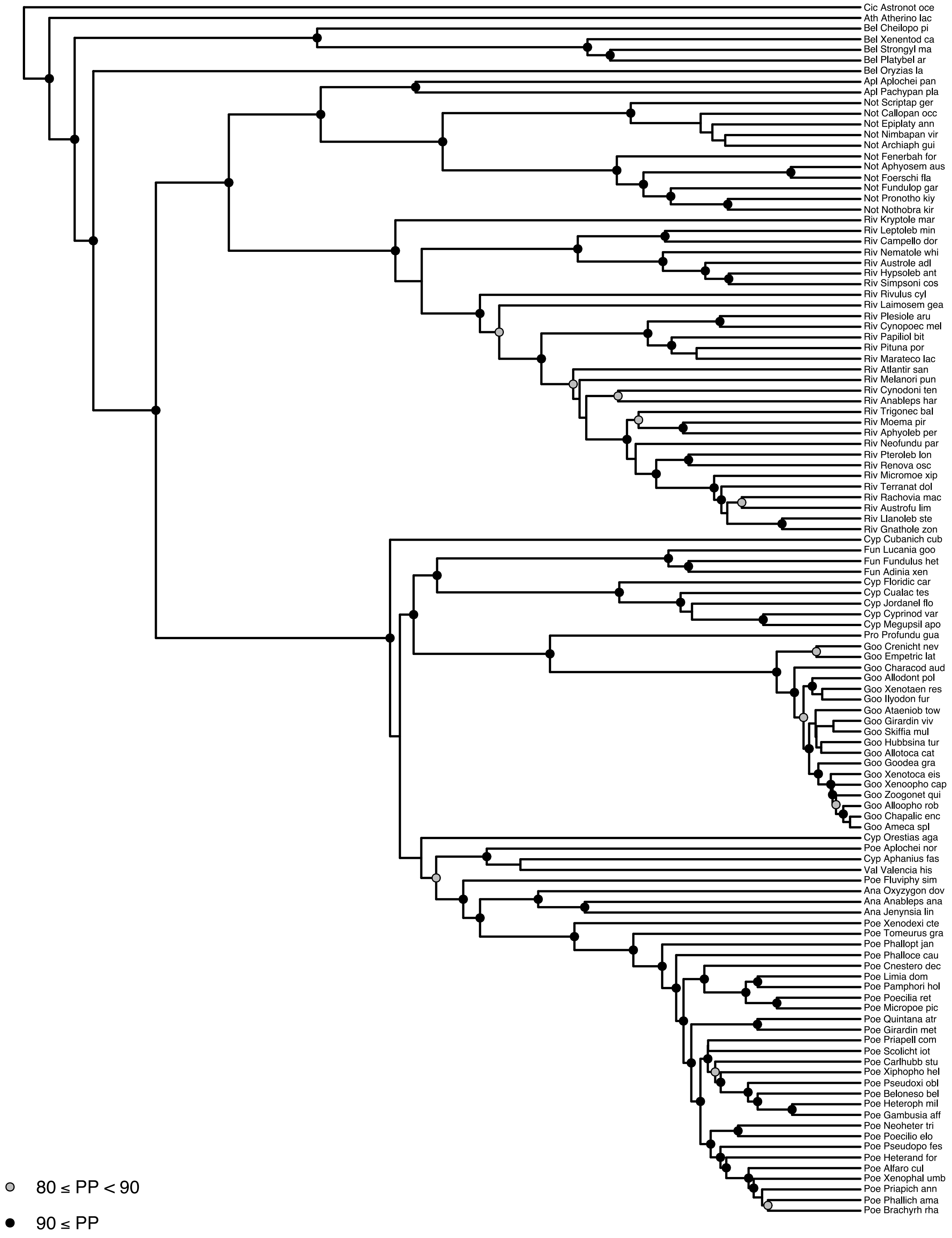

Supplementary Figure 2 | MCC phylogenetic tree. Posterior probability (PP) labelled on nodes. Black circles represent PP over 0.9, grey circles represent PP from 0.8-0.9, no circles represent PP under 0.8. Tip labels are abbreviated to Family_Genus_Species of the organism sequence data was taken from.

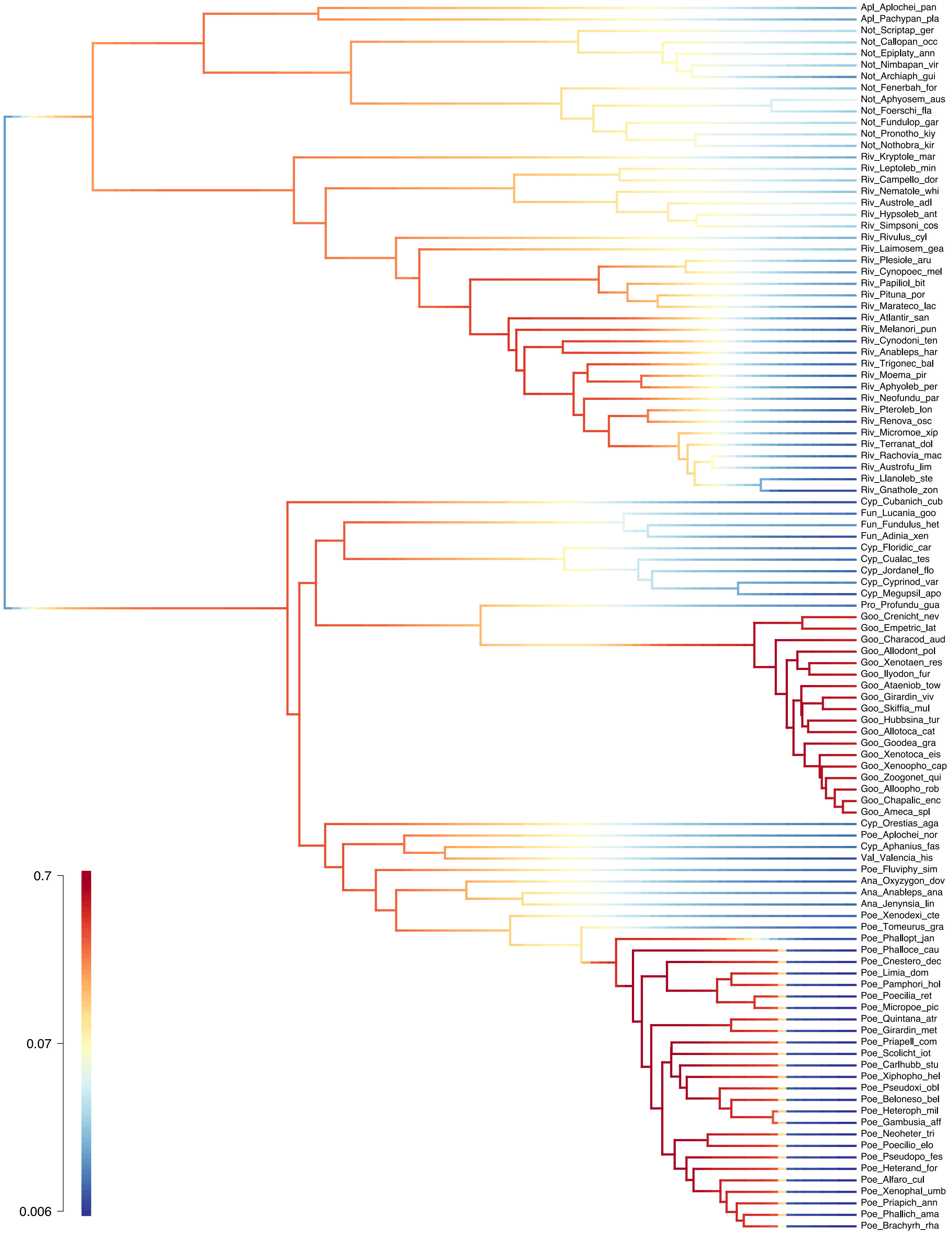

Supplementary Figure 3 | Mean phylorate plot of net diversification from BAMM analysis. Tip labels are abbreviated to Family_Genus_Species of the organism sequence data was taken from.

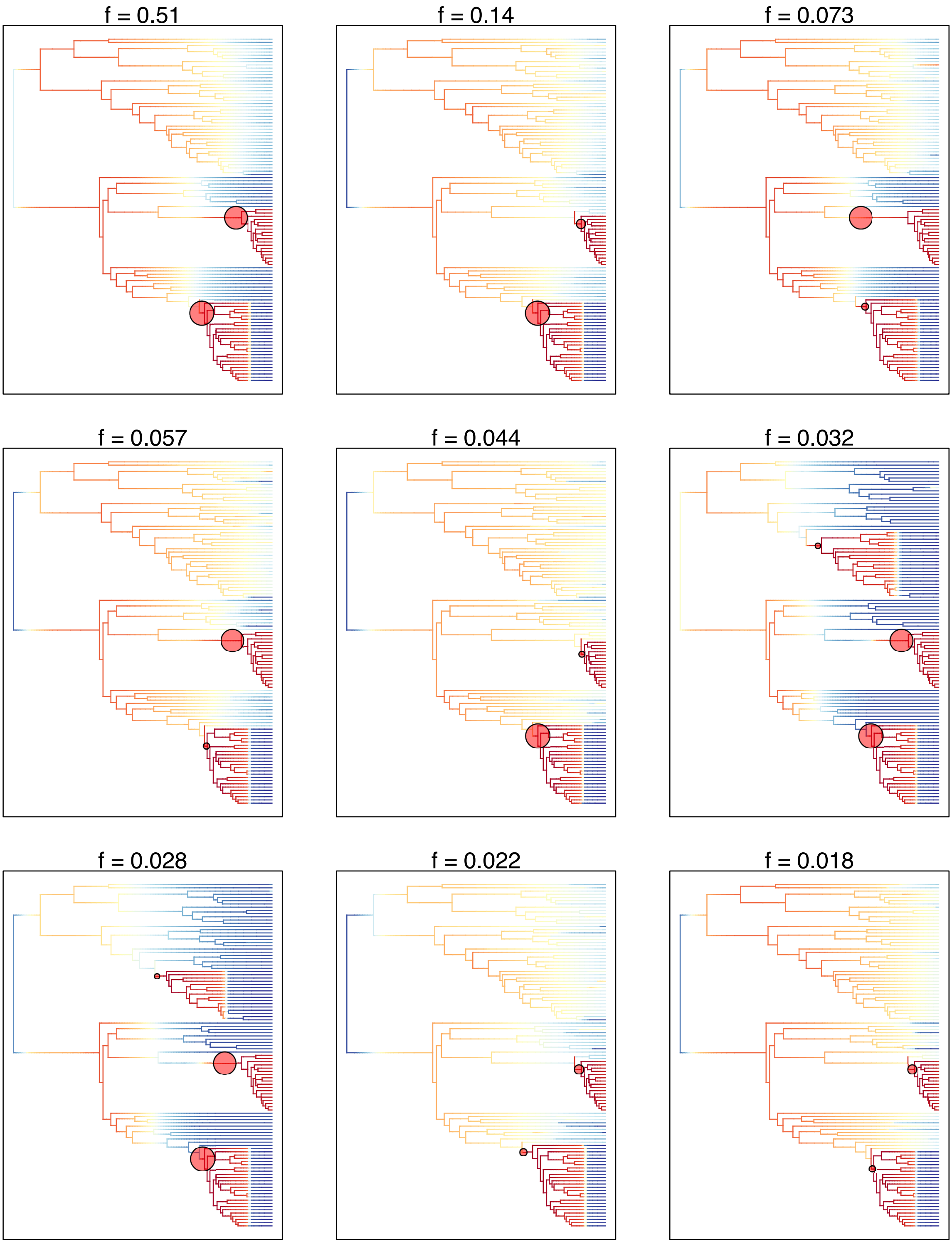

Supplementary Figure 4 | The nine most common Credible Shift Sets from the BAMM analysis. Red circles indicate where rate shifts take place. The size of the circle indicates the strength of the rate shift and the red colour indicates rate acceleration, blue a deceleration. ‘f’ is the posterior probability of each shift configuration.

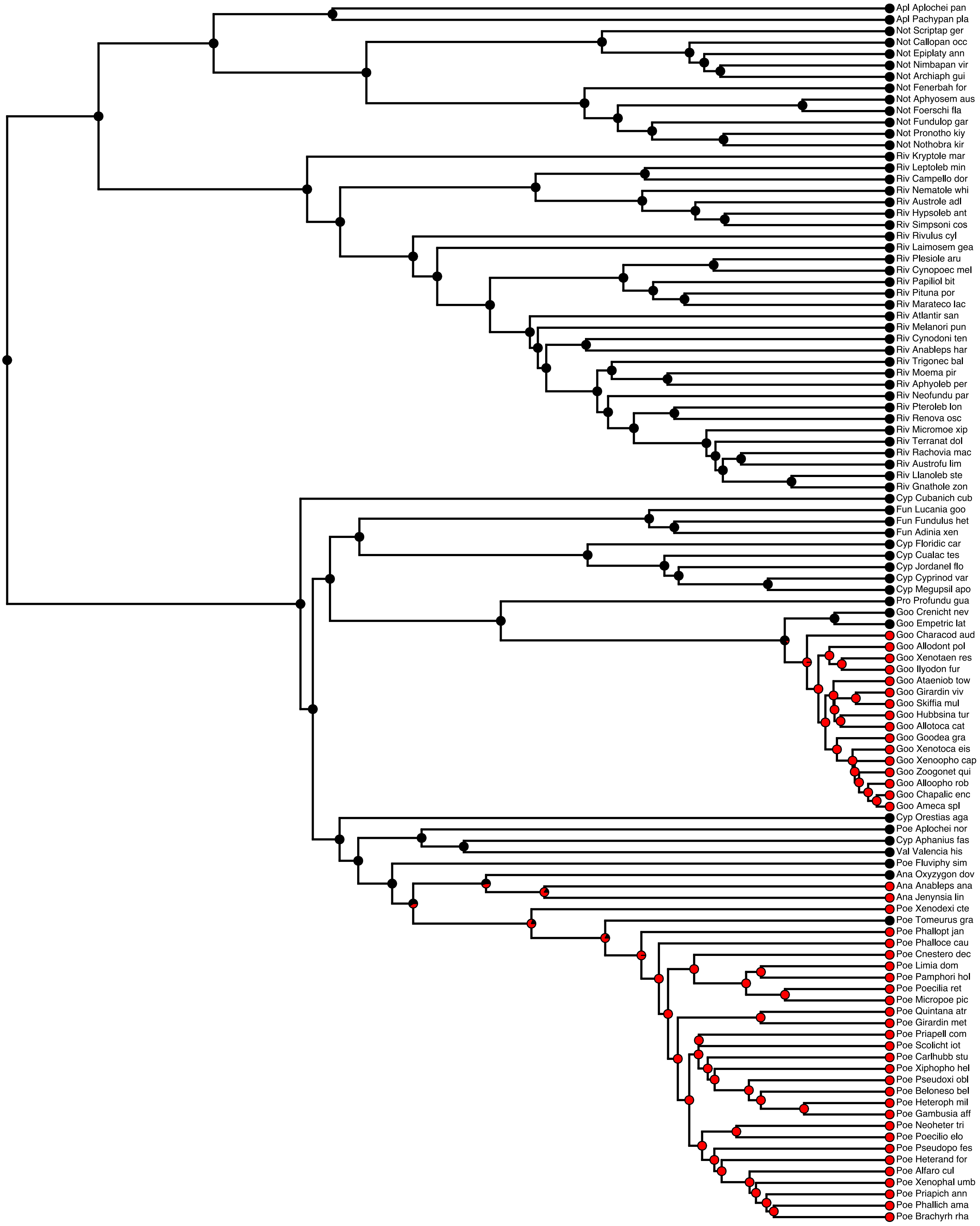

Supplementary Figure 5 | Ancestral state reconstructions of viviparity using stochastic character mapping. Red circles denote viviparity and black circles denote oviparity. Tip labels are abbreviated to Family_Genus_Species of the organism sequence data was taken from.

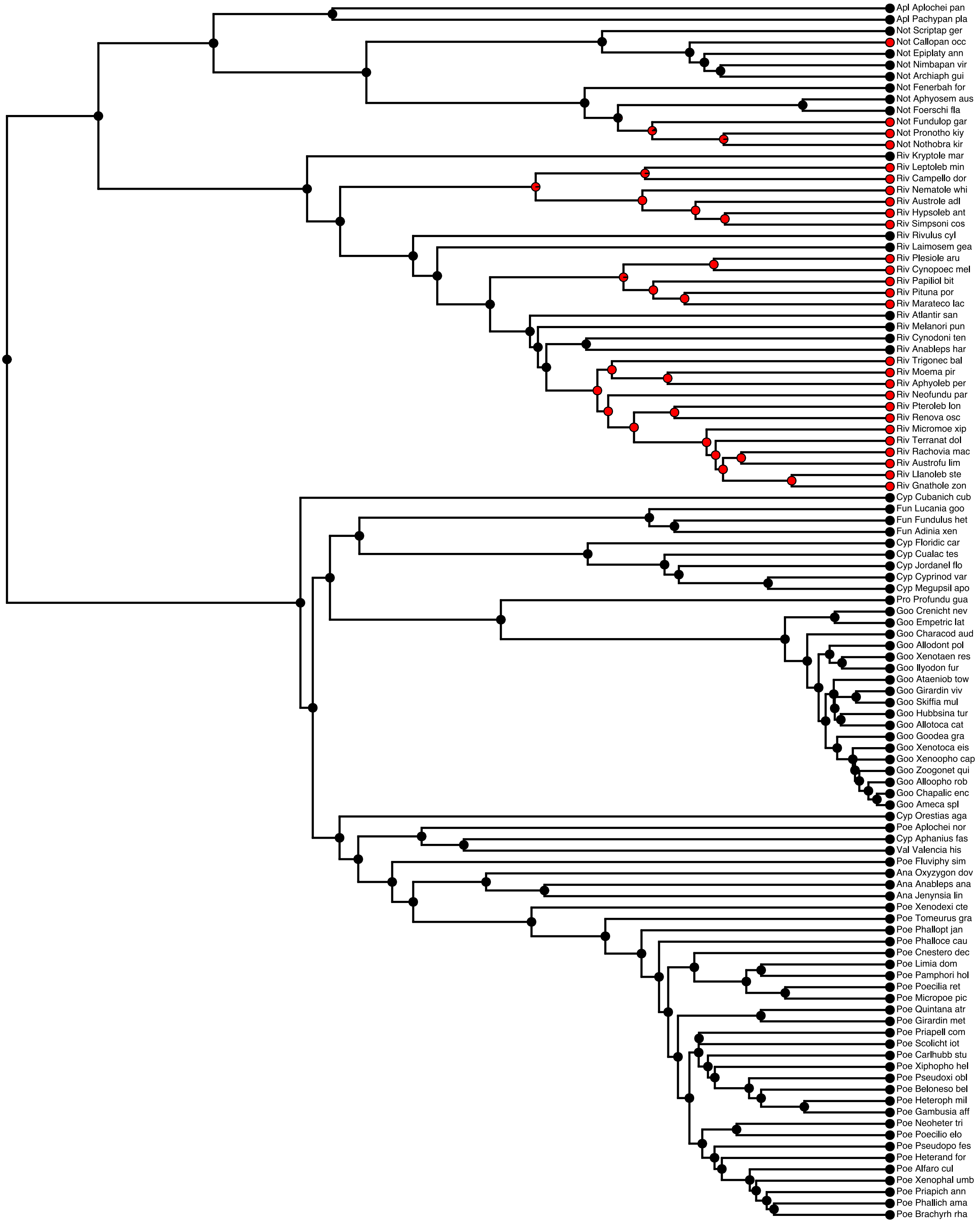

Supplementary Figure 6 | Ancestral state reconstructions of annualism using stochastic character mapping. Red circles denote annualism and black circles denote non-annualism. Tip labels are abbreviated to Family_Genus_Species of the organism sequence data was taken from.

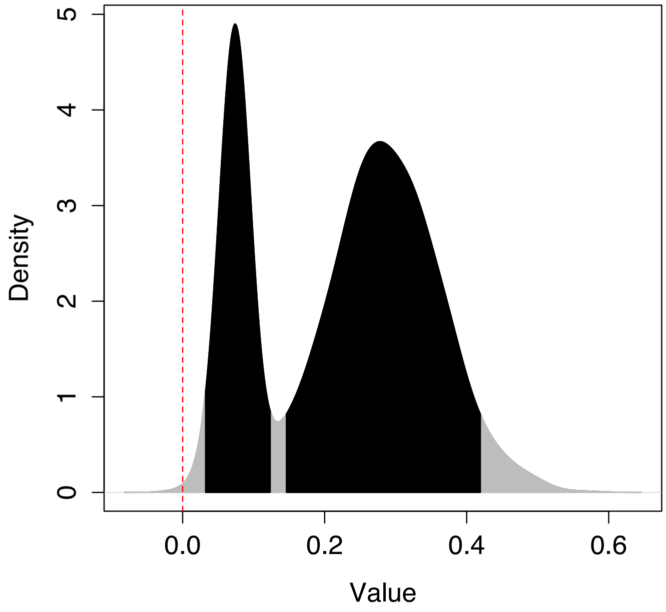

Supplementary Figure 7 | Credible interval of posterior samples from the oviparous goodeid subfamily Empetrichthyinae. Black regions represent 95% probability intervals, dotted vertical line marks 0.

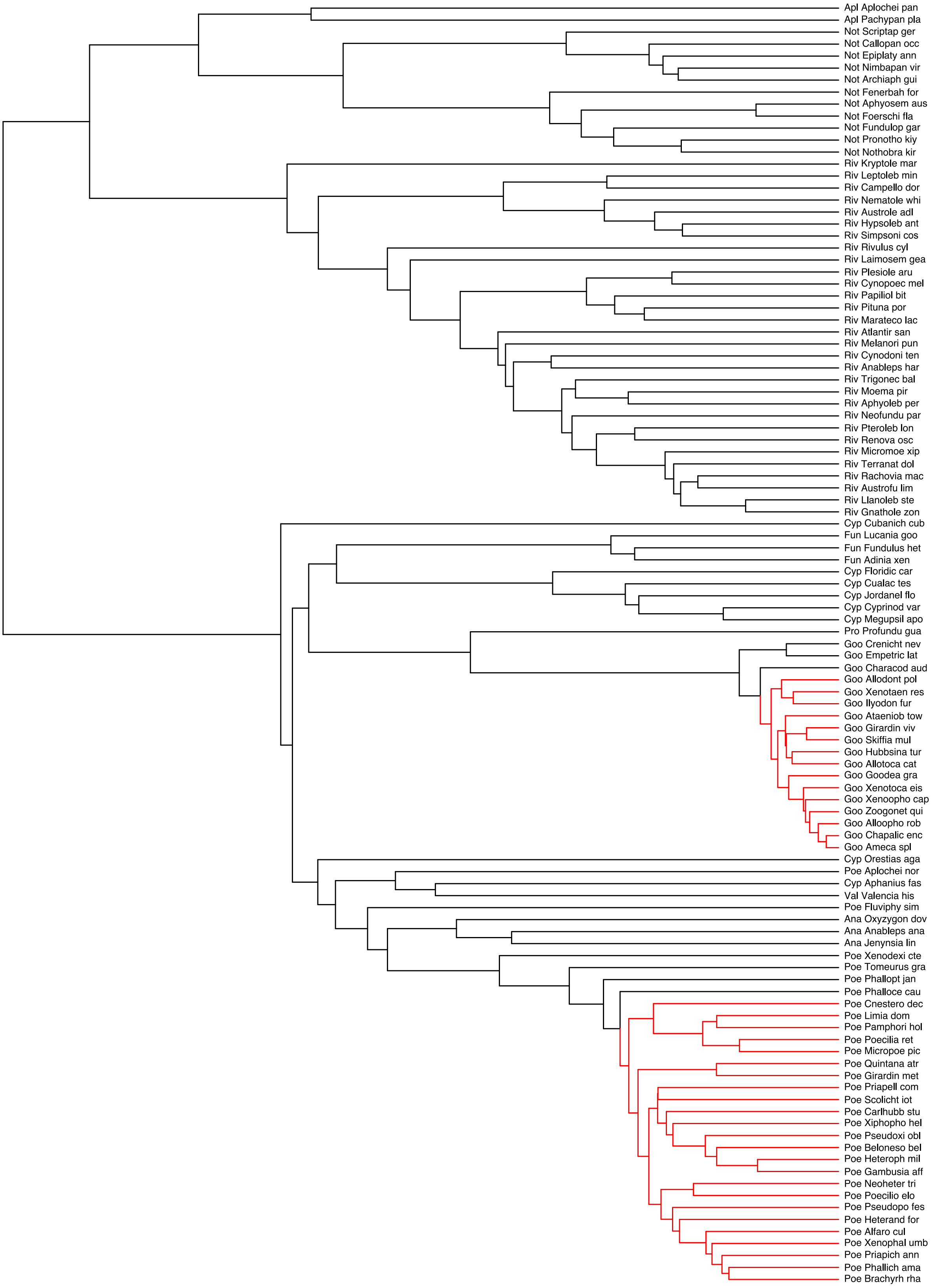

Supplementary Figure 8 | Cumulative shift probability. Branches in red indicate 95% posterior probability that a rate shift has occurred between that branch and the root of the tree. Tip labels are abbreviated to Family_Genus_Species of the organism sequence data was taken from.

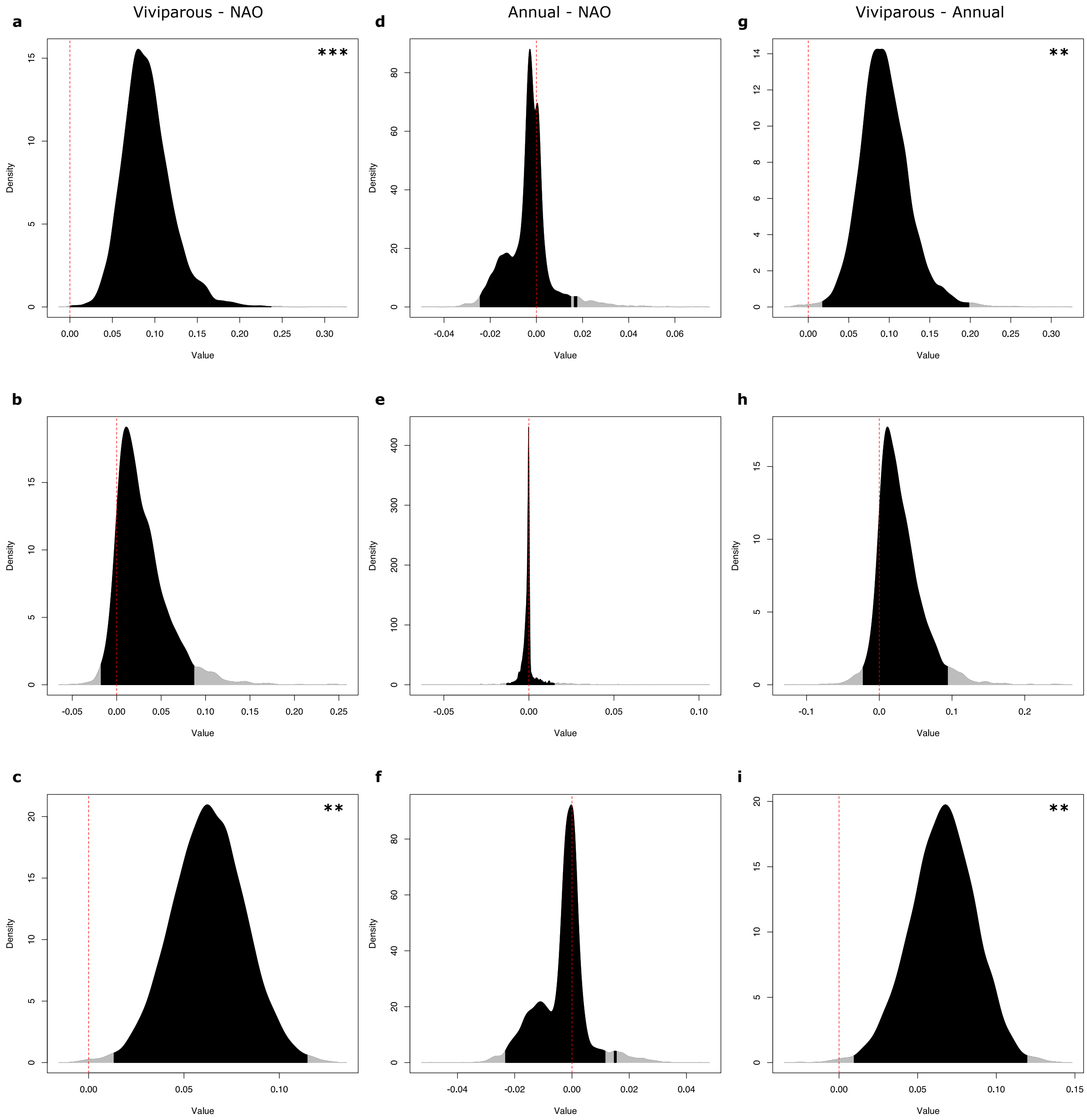

Supplementary Figure 9 | Credible intervals of differences from posterior distribution of samples taken from BAMM analyses. Graphs in the left column compare viviparous and non-annual oviparous groups for **a** speciation rate, **b** extinction rate and **c** net diversification rate. Graphs in the middle column compare **d** speciation rate, **e** extinction rate and **f** net diversification rate of annual and non-annual oviparous groups. Graphs in the right column compare **g** speciation rate, **h** extinction rate and **i** net diversification rate for viviparous and annual groups. Dotted red line indicates 0 difference between samples. Black regions represent probability intervals. Significance is calculated as the percentage of credible differences that do not overlap with zero, represented as * ≥ 95%, ** ≥ 99% and *** ≥ 99.9%; one-tailed.

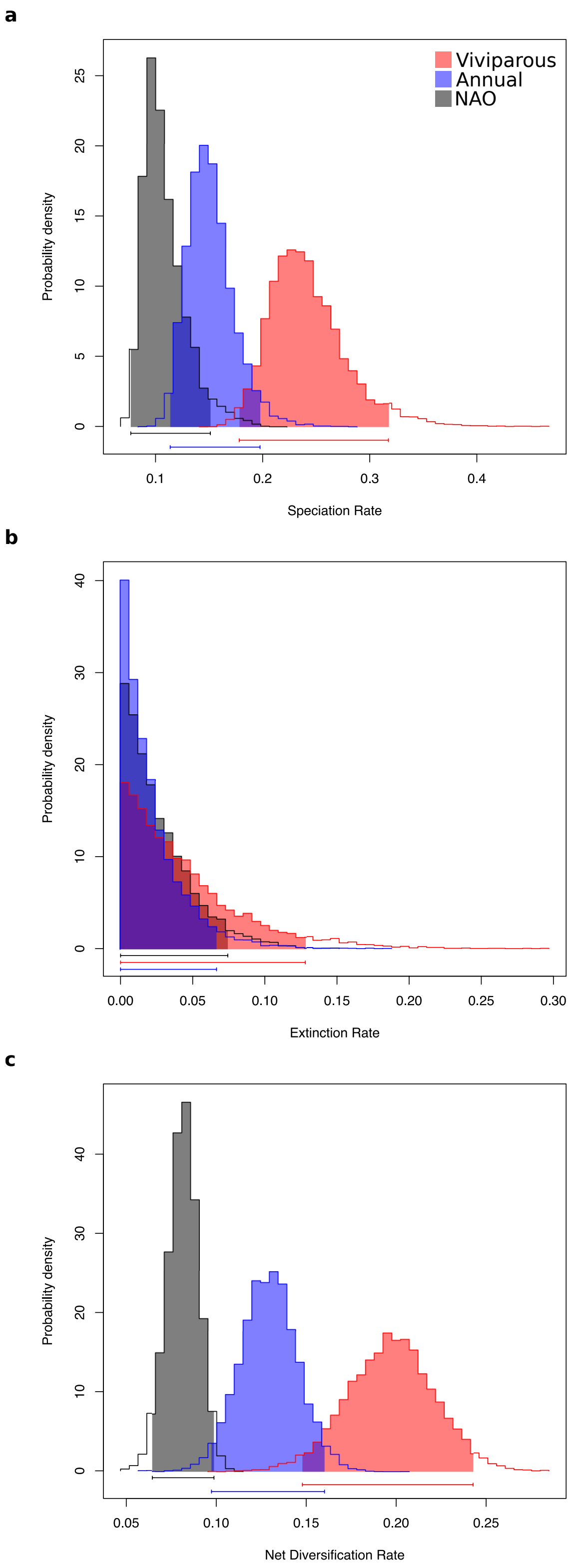

Supplementary Figure 10 | Posterior distribution of state-dependent rates. Rates are taken from a full 12 parameter MuSSE model. Graphs are separated into **a** speciation rate, **b** extinction rate and **c** net diversification rate for viviparous (red), annual (blue) and non-annual oviparous (NAO) (grey).

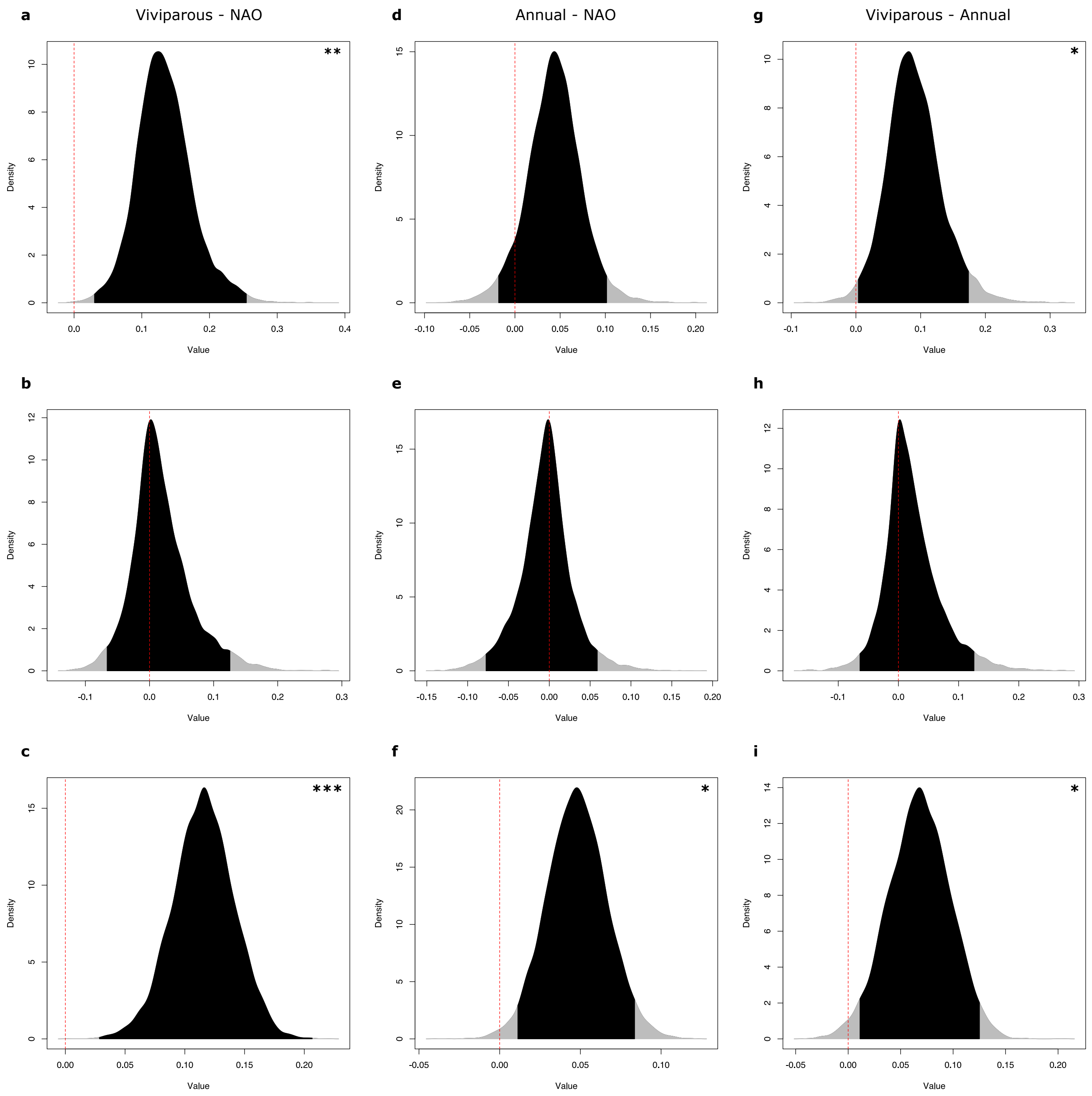

Supplementary Figure 11 | Credible intervals of differences from posterior distribution of samples taken from MuSSE analyses. Graphs in the left column compare viviparous and non-annual oviparous groups for **a** speciation rate, **b** extinction rate and **c** net diversification rate. Graphs in the middle column compare **d** speciation rate, **e** extinction rate and **f** net diversification rate of annual and non-annual oviparous groups. Graphs in the right column compare **g** speciation rate, **h** extinction rate and **i** net diversification rate for viviparous and annual groups. Dotted red line indicates 0 difference between samples. Black regions represent probability intervals. Significance is calculated as the percentage of credible differences that do not overlap with zero, represented as * ≥ 95%, ** ≥ 99% and *** ≥ 99.9%; one-tailed.

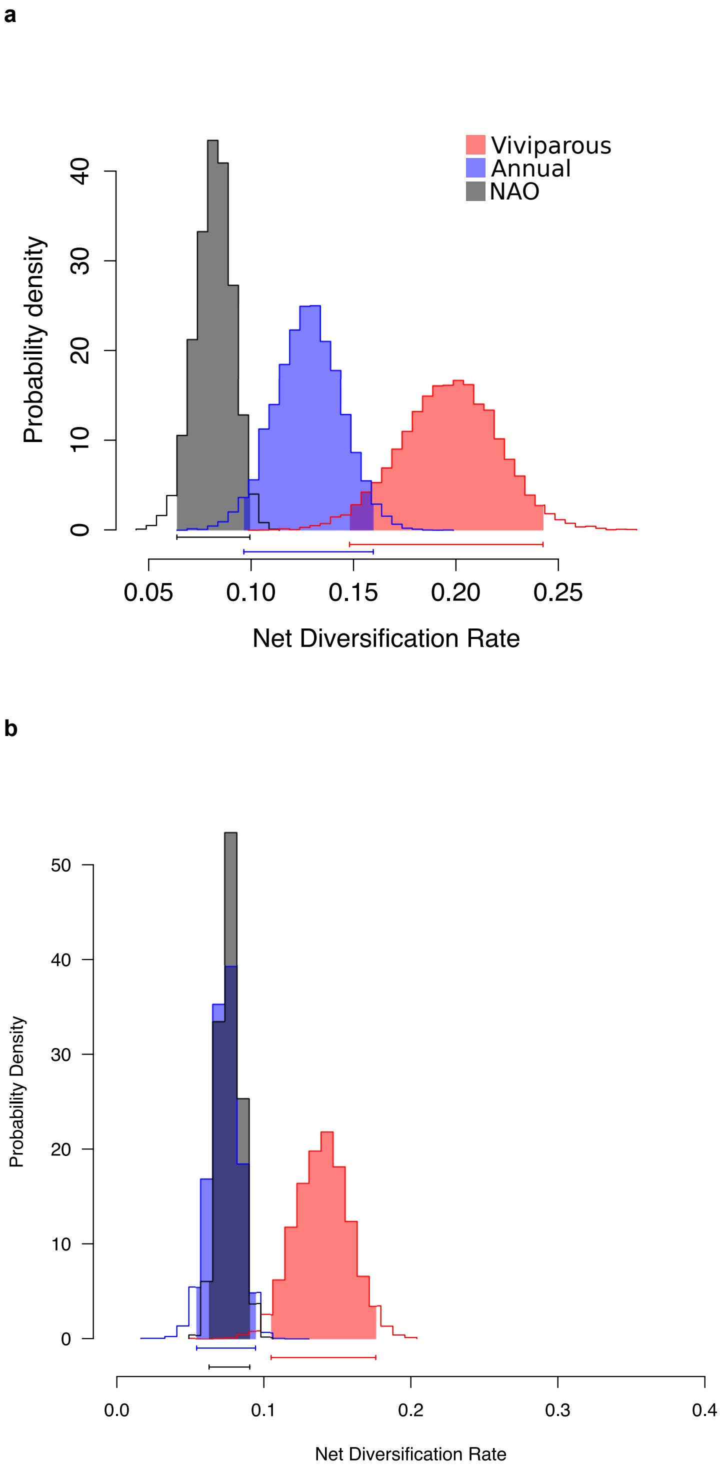

Supplementary Figure 12 | Posterior distribution of state-dependent rates. Rates taken from **a** MuSSE analyses and **b** BAMM analyses where the genus *Fundulopanchax* has been changed from annual to non-annual. Graphs show net diversification rate for viviparous (red), annual (blue) and non-annual oviparous (NAO) (grey).

Supplementary Table 1 | Fossils found and calibration parameters of those used. For normal priors, means and standard deviations were specified from the node ages and 95% highest posterior density of the tree in Betancur-R *et al.*[1] Wide, truncated priors (i.e. non-infinite) were used to ensure appropriate starting points for BEAST runs and minimize their effect on parameter estimation. Offsets for lognormal priors were set as the minimum age of the geological time period (e.g. epoch or age) to which each fossil was assigned. Mean values were set to the midpoint of geological time period and the standard deviation was set to one.

Node Label in Supplementary Fig. 1	Group	Mean (Ma)	Log (st dev)	Offset	Lower	Upper	Prior Distribution	Reference	Epoch/Age
A	Perciformes	97.3	7.48	-	37.3	157.3	Truncated Normal	1	-
B	Atheriniformes	77.4	11.6	-	37.4	117.4	Truncated Normal	1	-
C	Beloniformes	67.5	12.1	-	27.5	107.5	Truncated Normal	1	-
D	*Carrionellus* (Orestiini)	19.5	1	15.97	-	-	Lognormal	2	Lower Miocene
E	*Aphanius*	21.73	1	20.43	-	-	Lognormal	3	Aquitanian
F	*Fundulus*	17.015	1	13.6	-	-	Lognormal	4,5	Early Barstovian to late Hemingfordian
G	*Cyprinodon*	3.955	1	2.58	-	-	Lognormal	5,6	Pliocene
H	*Empetricthys*	3.955	1	2.58	-	-	Lognormal	5,7	Pliocene
-	*Prolebias**	-	-	-	-	-	-	8,9	Rupélien
-	*Aphanius*	-	-	-	-	-	-	3	Lower argonian
-	*Aphanius*	-	-	-	-	-	-	10	Messinian
-	*Aphanius*	-	-	-	-	-	-	11	Upper Micoene
-	*Aphanius*	-	-	-	-	-	-	12	Late Bessarabian to early Khersonian
-	*Fundulus*	-	-	-	-	-	-	5	Miocene
-	*Fundulus*	-	-	-	-	-	-	13	Middle Miocene
-	*Jenynsia***	-	-	-	-	-	-	14	Pleistocene
-	*Kenyaichthys****	-	-	-	-	-	-	15	Late Miocene

* There is uncertainty about which groups are the closest relatives of *Prolebias*. Costa[9] suggests that *Prolebias* is a *"paraphyletic assemblage, probably comprising taxa closely related to three distinct families, the Cyprinodontidae, the Valenciidae, and the Poeciliidae*."

** The *Jenynsia* fossil found corresponded to the Bonaerian Stage-Age (Late Middle Pleistocene), which is just 0.8-0.13 Ma and thus not likely to be informative for dating the divergence of *Jenynsia* and *Anableps*.

*** The fossil *Kenyaichthys* was placed as sister to all Rivulidae[15], which was unexpected and probably due to lack of available synapomorphies in the Rivulidae, Nothobranchiidae and Aplocheilidae[15]. The fossil is approximately 5.7-6 Ma, while the Rivulidae have already been found to be much older[16]. For these reasons, we deem the fossil to be unreliable for dating.

Supplementary References

1. Betancur-R, R. et al. The Tree of Life and a New Classification of Bony Fishes. PLoS Curr (2013). doi:10.1371/currents.tol.53ba26640df0ccaee75bb165c8c26288
2. Costa, W. J. E. M. Redescription and phylogenetic position of the fossil killifish† *Carrionellus diumortuus* White from the Lower Miocene of Ecuador (Teleostei: Cyprinodontiformes). Cybium 35, 181–187 (2011).
3. Reichenbacher, B. & Kowalke, T. Neogene and present-day zoogeography of killifishes (*Aphanius* and *Aphanolebias*) in the Mediterranean and Paratethys areas. Palaeogeography, Palaeoclimatology, Palaeoecology 281, 43–56 (2009).
4. Lugaski, T. *Fundulus lariversi*, a new Miocene fossil cyprinodont fish from Nevada. Wasmann J. Biol. 35, 203–211 (1977).
5. Smith, G. R. Late cenozoic freshwater fishes of North America. Annu. Rev. Ecol. Syst. (1981). doi:10.2307/2097109
6. Miller, R. R. Four new species of fossil cyprinodont fishes from eastern California. J. Wash. Acad. Sci 35, 315–321 (1945).
7. Uyeno, T. & Miller, R. R. Relationships of *Empetrichthys erdisi*, a Pliocene Cyprinodontid Fish from California, with Remarks on the Fundulinae and Cyprinodontinae. Copeia 1962, 520 (1962).
8. Gaudant, J. Révision de *Prolebias stenoura* Sauvage, 1874 du Stampien (= Rupélien) de Limagne (centre de la France), espèce type du genre *Prolebias* (poisson téléostéen, Cyprinodontiformes). Geodiversitas 34, 409–423 (2012).
9. Costa, W. J. E. M. The caudal skeleton of extant and fossil cyprinodontiform fishes (Teleostei: Atherinomorpha): comparative morphology and delimitation of phylogenetic characters. Vertebrate Zoology 62, 161–180 (2012).
10. Carnevale, G., Landini, W. & Sarti, G. Mare versus Lago-mare: marine fishes and the Mediterranean environment at the end of the Messinian Salinity Crisis. Journal of the Geological Society 163, 75–80 (2006).

11. Gaudant, J. *Aphanius persicus* (Priem, 1908) (Pisces, Teleostei, Cyprinodontidae): une nouvelle combinaison pour *Brachylebias persicus* Priem, 1908, du Miocène supérieur des environs de Tabriz (Iran). Geodiversitas 33, 347–356 (2011).

12. Vasilyan, D., Reichenbacher, B. & Carnevale, G. A new fossil *Aphanius* species from the Upper Miocene of Armenia (Eastern Paratethys). Paläontol. Z. 83, 511–519 (2009).

13. Livingston, T. D. & Dattilo, B. F. Middle Miocene lacustrine strata and fossil killifish in a volcanic setting: the rocks of Pavits Spring, Nevada Test Site, NYE County, Nevada. Geological Society of America Abstracts (2004).

14. Bogan, S., de los Reyes, M. L. & Cenizo, M. M. Primer registro del género *Jenynsia* Günther, 1866 (Teleostei: Cyprinodontiformes) en el Pleistoceno Medio tardío de la provincia de Buenos Aires (Argentina). Papéis Avulsos de Zoologia (São Paulo) 49, 81–86 (2008).

15. Altner, M. & Reichenbacher, B. †Kenyaichthyidae fam. nov. and †Kenyaichthys gen. nov. – First Record of a Fossil Aplocheiloid Killifish (Teleostei, Cyprinodontiformes). PLoS ONE 10, e0123056–41 (2015).

16. Furness, A. I., Reznick, D. N., Springer, M. S. & Meredith, R. W. Convergent evolution of alternative developmental trajectories associated with diapause in African and South American killifish. Proceedings of the Royal Society B: Biological Sciences 282, (2015).

17. Froese, R. and D. Pauly. Editors. 2015. FishBase.World Wide Web electronic publication. www.fishbase.org, version (09/2014).

18. Meyer, A. & Lydeard, C. The evolution of copulatory organs, internal fertilization, placentae and viviparity in killifishes (Cyprinodontiformes) inferred from a DNA phylogeny of the tyrosine kinase gene X-src. *Proceedings of the Royal Society B: Biological Sciences* **254,** 153–162 (1993).

19. Parenti, L. R. A phylogenetic and biogeographic analysis of cyprinodontiform fishes (Teleostei, Atherinomorpha). Bulletin of the AMNH; v. 168, article 4. (1981).

20. Webb, S. A. *et al*. Molecular phylogeny of the livebearing Goodeidae (Cyprinodontiformes). *Molecular Phylogenetics and Evolution* **30,** 527–544 (2004).

21. Sonnenberg, R. & Busch, E. Description of *Callopanchax sidibei* (Nothobranchiidae: Epiplateinae), a new species of killifish from southwestern Guinea, West Africa. *Bonn zoological Bulletin* (2010).

22. Wourms, J. P. The developmental biology of annual fishes. III. Pre‐embryonic and embryonic

diapause of variable duration in the eggs of annual fishes. Journal of Experimental Zoology 182, 389-414 (1972).

23. Hrbek, T., Seckinger, J. & Meyer, A. A phylogenetic and biogeographic perspective on the evolution of poeciliid fishes. *Molecular Phylogenetics and Evolution* **43,** 986–998 (2007).

24. Parenti, L. R., LoNostro, F. L. & Grier, H. J. Reproductive histology of *Tomeurus gracilis* Eigenmann, 1909 (Teleostei: Atherinomorpha: Poeciliidae) with comments on evolution of viviparity in atherinomorph fishes. *J. Morphol.* **271,** 1399–1406 (2010).

25. Reznick, D., Hrbek, T., Caura S., De Greef, J. & Roff, D. Life history of *Xenodexia ctenolepis*: implications for life history evolution in the family Poeciliidae. *Biological Journal of the Linnean Society* **92,** 77–85 (2007).

26. Domínguez-Castanedo, O. First observations of annualism in *Millerichthys robustus* (Cyprinodontiformes: Rivulidae). *Ichthyological Exploration of Freshwaters*, **24,** 15–20 (2013).
